# Nitrate and Nitrite Exposure Induces Visual Impairments in Adult Zebrafish

**DOI:** 10.3390/toxics12070518

**Published:** 2024-07-18

**Authors:** Febriyansyah Saputra, Mitsuyo Kishida, Shao-Yang Hu

**Affiliations:** 1 Graduate School of Science and Technology, Kumamoto University, Kumamoto 860-8555, Japan; ryansaputra252@gmail.com; 2 Department of Biological Science and Technology, National Pingtung University of Science and Technology, Pingtung 912, Taiwan

**Keywords:** nitrate, nitrite, visual impairment, zebrafish, environmental pollutants

## Abstract

Nitrate and nitrite have emerged as increasingly common environmental pollutants, posing significant risks to various forms of life within ecosystems. To understand their impact on the visual system of zebrafish, adult zebrafish were exposed to environmentally relevant concentrations of nitrate (10 mg/L) and nitrite (1 mg/L) for 7 days. Visual behaviors were examined using optomotor and avoidance response. The eyeballs of the zebrafish were collected for H&E staining, IHC, and qPCR. Exposure decreased visual behavior and the thickness of most retinal layers. Exposure decreased expression of *pax6a*, *pax6b*, *gpx1a*, and *bcl2a*. Exposure increased expression of *esr1*, *esr1a*, *esr2b*, *cyp19a1b*, *sod1a*, *nos2a*, *casps3*, and *tp53*, and increased retinal brain aromatase expression by IHC. Collectively, our findings demonstrate that nitrate and nitrite exposure negatively impacted the visual system of adult zebrafish, highlighting the potential hazards of these environmental pollutants on aquatic organisms.

## 1. Introduction

As industrialization, urbanization, and agricultural modernization have advanced in society, human-induced groundwater pollution has significantly increased [1]. Nitrate emerges as a significant water contaminant, seeping into water reservoirs as a result of human activities such as livestock farming, agricultural fertilization practices, and industrial effluent discharge. This compound then infiltrates surface and groundwater directly through runoff, polluting these water bodies [2]. Human activities are the main source of nitrogen in the ecosystem, primarily through the use of fertilizers. Approximately half of these fertilizers seep into surface and groundwater, leading to increased nitrate levels in water bodies. Nitrate pollution is now a significant concern, ranking as the second most significant chemical contaminant in surface and groundwater alongside pesticides [3]. The challenge with nitrate contamination lies in its various forms, such as ammonia, nitrite, atmospheric nitrogen, and bound nitrogen [4]. Nitrate has the ability to endure in groundwater for extended periods and can accumulate to elevated concentrations over time. Many dangerous and possibly carcinogenic compounds have been detected in the groundwater nearby, with nitrate being especially hazardous [5].

The World Health Organization (WHO) has set safe levels for nitrate and nitrite in drinking water to prevent immediate health issues, like methemoglobinemia and thyroid effects [6]. Unfortunately, these safe limits are often surpassed in many countries, particularly in shallow waters and wells, regardless of their development status [7]. Elevated nitrate concentrations in environments exceed 25 mg/L NO_3_-N in polluted surface waters and 100 mg/L NO_3_-N in contaminated groundwaters [8]. A growing body of observational research has revealed links between nitrate levels in drinking water and various human diseases, which encompass distinct forms of cancer [9]. Despite the increasing global awareness regarding elevated nitrate concentrations in ground and surface waters, research on the toxicity of nitrates to aquatic organisms remains limited. One of the main detrimental impacts of nitrates on aquatic animals is their capability to transform oxygen-carrying pigments such as hemoglobin and hemocyanin into inactive forms that are unable to transport oxygen, such as methemoglobin [10]. However, due to the limited uptake of nitrate compared to other nitrogen compounds like ammonia and nitrite, which have higher branchial permeability, nitrate’s toxicity to aquatic animals is relatively low [11,12]. Studies in zebrafish have demonstrated that significantly higher concentrations (100–300 mg/L) result in developmental defects, growth suppression, and high mortality [13,14]. While some studies have suggested adverse effects of nitrate and nitrite exposure on the nervous system [15] and dopamine neurons [16], as well as its potential to induce mild anxiogenic-like behavior and alter brain metabolomic profiles [17].

It is now well established that the anions NO_3_^−^ and NO_2_^−^ can be converted to NO in blood and tissues [18]. This conversion occurs through both enzymatic and non-enzymatic pathways, providing a significant source of NO, especially under conditions where the synthesis of NO through nitric oxide synthase (NOS) is impaired [19]. NO is a crucial signaling molecule involved in numerous physiological processes, including the regulation of cardiovascular function, neurotransmission in the central nervous system, and the modulation of immune responses [20,21]. Through enzymatic pathways, such as those involving xanthine oxidoreductase, as well as non-enzymatic pathways like acidic reduction in the stomach, nitrate and nitrite can be reduced back to NO, thereby replenishing NO levels in the body [19]. This multifaceted involvement of NO underscores the importance of its continuous availability and the role of nitrate and nitrite as reservoirs for NO bioactivity.

Zebrafish, also known as *Danio rerio*, are widely used as a vertebrate model in various scientific disciplines such as endocrinology, toxicology, developmental biology, and vision studies [22]. Zebrafish genome sequencing has made it highly valuable for researching various disorders and diseases, boasting over 26,000 protein-coding genes, with 70% having clear counterparts in humans [23]. Additionally, zebrafish are a valuable model organism for investigating visual system development, function, and disease mechanisms due to their technical advantages. Their eyes exhibit similarities with humans in anatomy, circuitry, physiology, and gene expression, enhancing their utility in research [24]. The zebrafish retina exhibits comparable cell types and circuitry to the human retina, allowing for the modeling of specific retina-related diseases observed in humans, including red color blindness and congenital stationary night blindness [25].

This study is designed to thoroughly investigate the potential negative impacts of exposure to nitrate and nitrite on the visual system of zebrafish. To achieve this objective, we are employing a multidimensional approach that includes behavioral analysis, histological examinations, and molecular investigations. Through our research, we endeavor to shed light on the potential negative consequences associated with nitrate and nitrite exposure in relation to visual function. This investigation will contribute valuable insights into the effects of these environmental factors on visual health, thereby addressing an important aspect of aquatic organism well-being and environmental safety.

## 2. Materials and Methods

### 2.1. Animals

The study used adult wild-strain zebrafish (*Danio rerio*) aged 6 to 8 months. The average body weight was 0.67 ± 0.03 g (mean ± SD), while the total length was 3.05 ± 0.18 cm (mean ± SD). The zebrafish were reared in 10 L tanks with continuous water exchange using dechlorinated tap water (refreshed at 5% daily) at a temperature of 28 °C. The light regime consisted of 14 h of light followed by 10 h of darkness, and the fish were fed twice daily with commercial pellets. The pH level of the water was regularly monitored and maintained at 7.0. All procedures and care for the fish were performed according to Taiwan’s rules on animal welfare and the Association for Assessment and Accreditation of Laboratory Animal Care International’s (AAALAC) guidelines.

### 2.2. Nitrate and Nitrite Exposure

Sodium nitrate (Wako) and sodium nitrite (Wako) were dissolved in distilled water to achieve concentrations of 10 mg/L nitrate–nitrogen (NO_3_-N) and 1 mg/L nitrite–nitrogen (NO_2_-N), respectively. Adult zebrafish were exposed to three different concentrations for 7 days: control group, 10 mg/L nitrate-exposed group, and 1 mg/L nitrite-exposed group. These concentrations was chosen based on regulatory limits or guidelines set by environmental agencies (EPA) and the WHO [6]. Previous research have shown that these levels altered zebrafish dopaminergic neurons [16]. The fish were randomly allocated into tanks containing 1.5 L of the respective solutions, with 10 fish per tank (triplicate). Water quality parameters, including temperature, ammonia, nitrate, nitrite, and pH, were measured twice daily using the LAQUAtwin kit: morning at 9:00 pre-feeding and afternoon at 17:00 post-feeding for 7 days (Table 1). To maintain consistent concentrations and water quality, the tank water was changed daily. Throughout the experiment, the fish were fed commercial pellets daily.

### 2.3. Optomotor Response (OMR)

**Figure 1:**
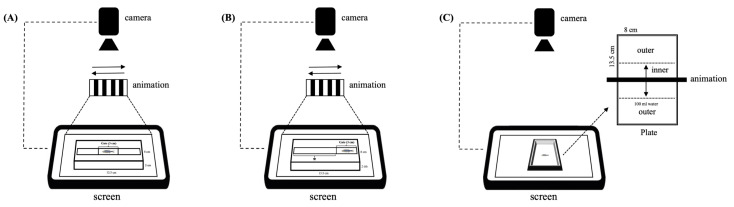
Instruments for visual behavior assessment. (**A**) OMR positive response measurement. (**B**) OMR swimming distance measurement (* indicates swimming distance). (**C**) Avoidance behavior measurement. The direction of the animation is indicated by arrows (→). The experiments were carried out at room temperature in a dark place.

**Figure 2:**
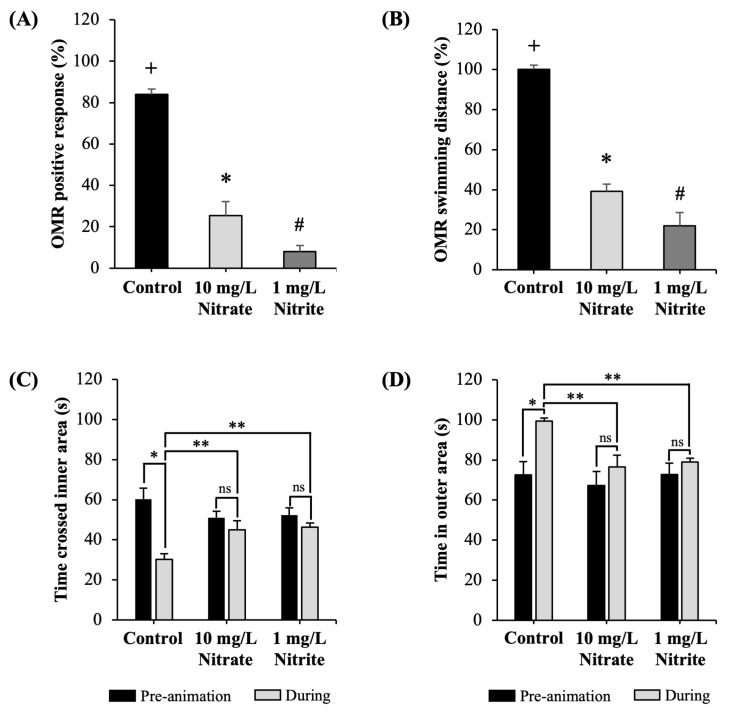
Effects of nitrate and nitrite in the visual behavior responses. Effects on OMR response in adult zebrafish: (**A**) positive OMR responses; (**B**) positive OMR swimming distance. Each value is expressed as the mean ± SD (*n* = 5 fish). Different marks in each graph (+, *, #) indicate significant differences (*p* < 0.05). Effects on avoidance behavior: (**C**) time crossing inner area; (**D**) time in outer area. Each value is expressed as the mean ± SD (*n* = 5 fish). Symbols indicate statistical significance (* *p* < 0.05 compared to control group during pre-animation, ** *p* < 0.05 compared to the control group during animation; ns—not significant). Similar experiments were replicated three times.

OMR was utilized as a method to assess visual behavior in this study. OMR, which involves the organism turning its head or body in response to visual stimuli, is an effective tool for detecting abnormalities in visual function [26]. This method was adapted from the previous study Saputra et al. (2024) [27]. During the experiment, adult zebrafish were placed and confined within the gate of a specially designed track (1.5 cm × 13.5 cm with 30 mL water), either at the center or on the side. The fish were incubated to a white screen for 30 s with light intensity at 825 nm. Following this, a stimulus with leftward and rightward animations of white and black bars was presented. After 5 s of animation, the gate confining the fish was opened, and their response was recorded for 30 s. The OMR assay was evaluated based on two distinct behavior responses of the fish: (1) positive behavior (+), i.e., number of fish swimming in the same direction as the grating movement, indicating a sensitive OMR (Figure 1A); (2) swimming distance, i.e., the overall distance covered by the fish during the positive OMR from the start to the end of the track (see Figure 1B). During leftward animation of the OMR swimming distance, the gate was positioned on the right; conversely, during rightward animation, the gate was positioned on the left. The distance the fish swam from the gate to the end of the track was recorded and measured using ImageJ 1.53i software. Each group was examined using five fish, and the experiments were conducted three times.

### 2.4. Avoidance of Visual Stimulus

Avoidance behavior in response to visual stimuli was assessed in adult zebrafish as part of this study. This method was adapted based on recent published papers [28]. Individual zebrafish were randomly placed in a tank positioned on top of a horizontally arranged laptop screen. The visual stimulus was generated using Microsoft PowerPoint on the laptop, consisting of a single black rectangle (1 cm × 29 cm). This rectangle repeatedly oscillated forward and backward within inner zones located at the center of the tank. The experimental procedure began by allowing the zebrafish to acclimate on a white screen for 2 min in a designed plate (8 cm × 13.5 cm) containing 100 mL of tap water (Figure 1C). Subsequently, the visual stimulus animation was presented for 2 min, and the fish responses were recorded. During analysis, the time spent by the fish in the outer zone and the frequency of crossings into the inner area were quantified both before and during the animation. Each group was examined using five fish, and similar experiments were replicated three times.

### 2.5. Histological Analysis

The eyes of adult zebrafish were fixed in 4% paraformaldehyde following 7 days of exposure to nitrate and nitrite. They underwent dehydration using ascending ethanol concentrations as outlined in the procedure by Sullivan-Brown et al. (2011) [29]. Subsequently, the fixed eyes were embedded in paraffin. Thin tissue slices measuring 8 μm in thickness were prepared and stained using hematoxylin and eosin (H&E) staining. The stained tissue sections were then photographed at a magnification of ×400 using a fluorescence microscope (Olympus BX51, Tokyo, Japan). The investigation of retina layers followed the procedure outlined by Caioni et al. (2023) [24]. Each group was examined using five fish, and the experiments were conducted three times.

### 2.6. Immunohistochemistry (IHC)

In the immunohistochemistry analysis, a rabbit antibody targeting zebrafish brain aromatase (*AromB*) (Sigma-Genosys, Woodlands, TX, USA) was employed. Slides were deparaffinized and blocked, after which they were subjected to antibody staining using the primary antibody, rabbit anti-AroB (1:200). For chromogenic immunodetection, an AP-conjugated secondary antibody, goat anti-rabbit IgG (1:1000, Abcam, Cambridge, UK), was applied, and the samples were observed under a light microscope. The immunohistochemistry-positive areas were quantitatively analyzed using the ImageJ software. Each group was examined using five fish, and the experiments were conducted three times.

### 2.7. Real-Time PCR

In this study, total RNA was extracted from the eyes of zebrafish exposed to nitrate and nitrite (*n* = 5 fish) using TRI reagent (Roche, Mannheim, Germany) following the manufacturer’s protocol. The reverse transcription of about 1 μg of total RNA was performed using an iScript cDNA Synthesis Kit (Bio-Rad, Foster, CA, USA) following the instructions provided by the manufacturer. The specific primers used in this experiment are outlined in Table 2. Real-time PCR analysis was performed using KAPA SYBR FAST PCR reagent and an Applied Biosystems StepOnePlus Real-Time PCR system. In the real-time PCR system, enzyme activation at 95 °C for 3 min was followed by 40 cycles of denaturation at 95 °C for 3 s and annealing and extension at 60 °C for 20 s. Relative expression levels were normalized by a housekeeping gene, *eef1a1*.

### 2.8. Statistical Analysis

The experiments were conducted in triplicate. The data were first evaluated for normality and homogeneity using Bartlett’s test. Afterwards, a unidirectional analysis of variance (ANOVA) was conducted, followed by Tukey’s post hoc test, to detect significant differences between the treatment groups and the control group. The statistical analyses were performed using the SigmaPlot 12.5 software package for Windows, with a significance level of *p* < 0.05 applied to all analyses. The data were presented as the mean ± standard deviation (SD).

## 3. Results

### 3.1. Effects on Visual Behavior Responses

To assess the potential impact of nitrate and nitrite on visual function, we conducted examinations of the OMR and avoidance behavior. The results showed that exposure to nitrate and nitrite significantly reduced positive behavior in response to the grating movement during the OMR test, indicating that the fish did not follow the direction of the grating movement as effectively as the control group, as shown in Figure 2A. Additionally, exposure resulted in a significant reduction in the swimming distance of zebrafish during the positive OMR (Figure 2B).

In cases of avoidance behavior, we measured the time spent in the outer zone and the time spent crossing the inner area before and during the animation. Interestingly, nitrate and nitrite exposure did not elicit a response in terms of avoidance behavior. In contrast, the control group of fish demonstrated a significant response to the avoidance stimulus, as evidenced by increased time spent in the outer zones and reduced time spent crossing the inner area (Figure 2C,D, respectively).

### 3.2. Effects on Retina Layers

**Figure 3:**
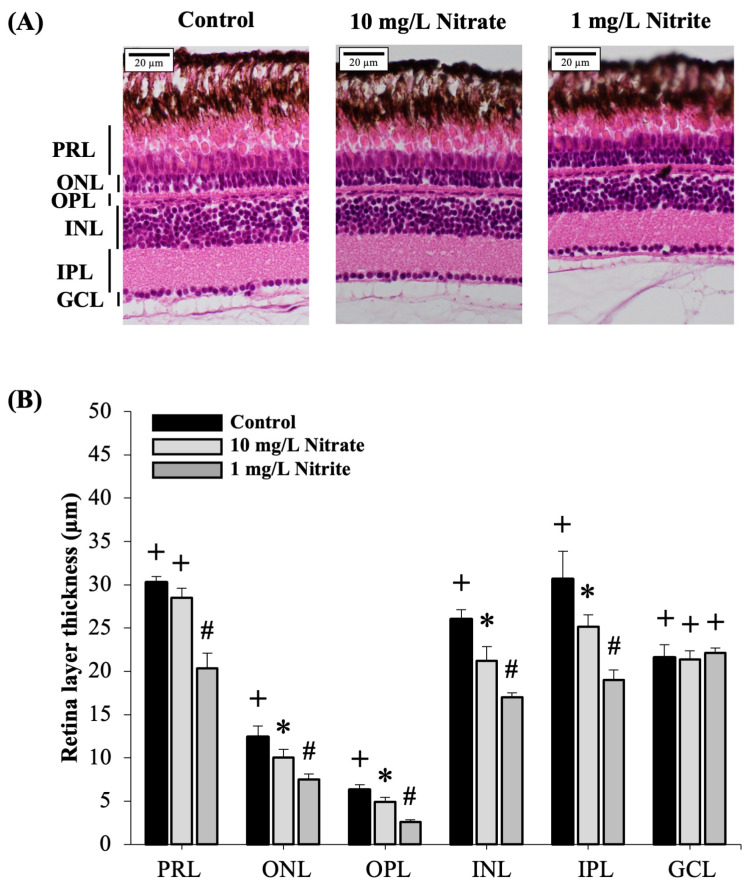
Effects of nitrate and nitrite exposure on zebrafish retina thickness was assessed through H&E staining. (**A**) Representative image of zebrafish retina exposed to control, 10 mg/L nitrate and 1 mg/L nitrite. (**B**) Retina layer thickness. PRL, photoreceptor layers; ONL, outer nuclear layer; OPL, outer plexiform layer; INL, inner nuclear cell layer; IPL, inner plexiform layer; GCL, ganglion cell layer. Each value is expressed as the mean ± SD (*n* = 5 fish). Different marks in each graph (+, *, #) indicate significant differences (*p* < 0.05). Similar experiments were replicated three times.

The concentric layers of the zebrafish retina, including the photoreceptor layers (PRL), outer nuclear layer (ONL), outer plexiform layer (OPL), inner nuclear cell layer (INL), inner plexiform layer (IPL), and ganglion cell layer (GCL), were clearly distinguishable in both the control and exposure groups (refer to Figure 3A). Our study demonstrated a significant reduction in overall retinal thickness in the nitrate and nitrite exposure group compared to the control group (see Figure 3B).

### 3.3. Effect on the Gene Expression

**Figure 4:**
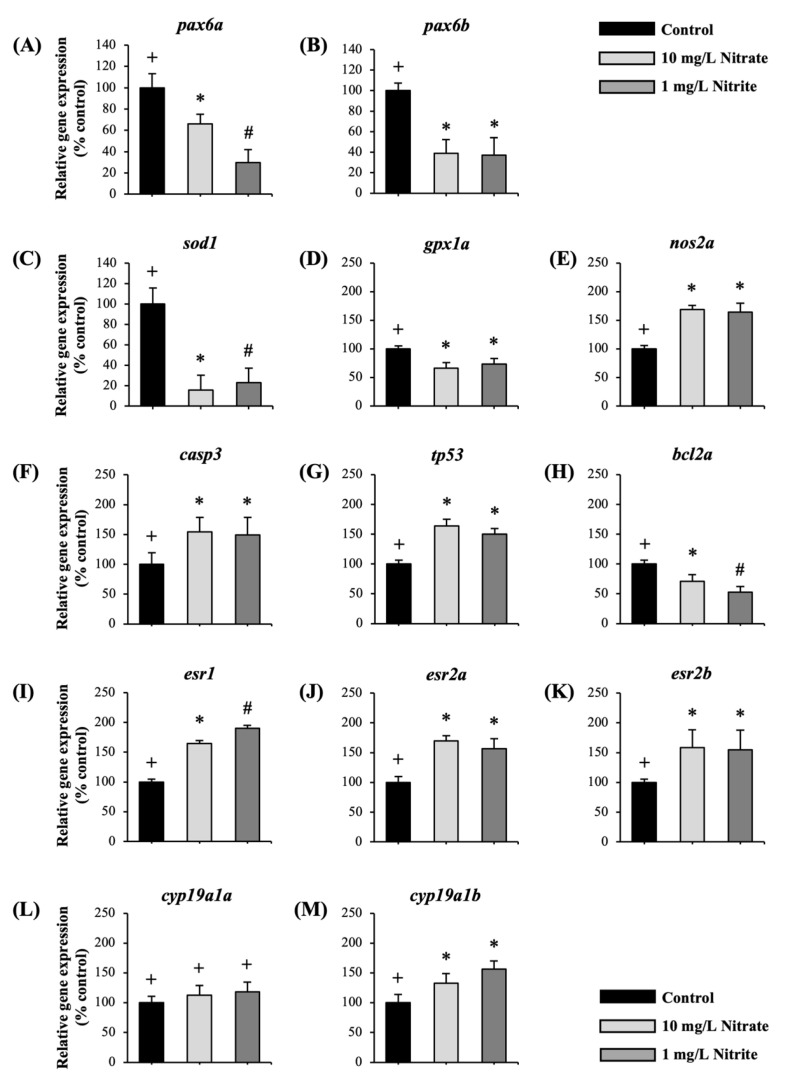
The effects of nitrate and nitrite exposure on relative gene expression in adult zebrafish eye. The impact of nitrate and nitrite exposure on relative gene expression was assessed using real-time PCR. Adult zebrafish were exposed to control, 10 mg/L nitrate, and 1 mg/L nitrite (*n* = 5 per treatment group). Relative expressions of genes related to various biological pathways were analyzed: (**A**,**B**) genes related to eye development; (**C**–**E**) genes related to oxidative stress; (**F**–**H**) genes related to apoptosis; (**I**–**K**) genes related to estrogen receptors (ERs); (**L**,**M**) genes related to aromatases. Each value is expressed as the mean ± SD. Different symbols (+, *, #) in each graph indicate significant differences (*p* < 0.05). Similar experiments were replicated three times.

In order to clarify the potential mechanisms responsible for the impairment of the zebrafish visual system caused by exposure to nitrate and nitrite, we investigated several key genes involved in eye function as *pax6a* and *pax6b*, oxidative-stress-associated genes including, *gpx1a*, *sod1a* and *nos2a*, apoptosis-related genes such as *casp3*, *tp53*, and *bcl2a*, estrogen receptor-related genes (*esr1*, *esr2a*, and *esr2b*), and ovarian and brain aromatases (*cyp19a1a* and *cyp19a1b*, respectively). The results of this study demonstrated a significant reduction in the expression of *pax6a* and *pax6b* genes following nitrate and nitrite exposure, as depicted in Figure 4A,B, respectively.

Furthermore, the expression of genes *sod1* (Figure 4C) *gpx1a* (Figure 4D) was reduced as a result of exposure to nitrate and nitrite, whereas the expression of *nos2a* (Figure 4E) was increased. The exposure resulted in increased gene expression of *casp3* (Figure 4F) and *tp53* (Figure 4G), as well as a decreased in the expression of *bcl2a* (Figure 4H). Significantly, exposure to nitrate and nitrite increased the expression of estrogen receptors *esr1* (Figure 4I), *esr2a* (Figure 4J), and *esr2b* (Figure 4K). In addition, although the expression of *cyp19a1a* did not alter after exposure, there was a significant increase in the expression of *cyp19a1b*, as seen in Figure 4L,M, respectively.

### 3.4. Effects on Brain Aromatase Protein in the Retina

**Figure 5:**
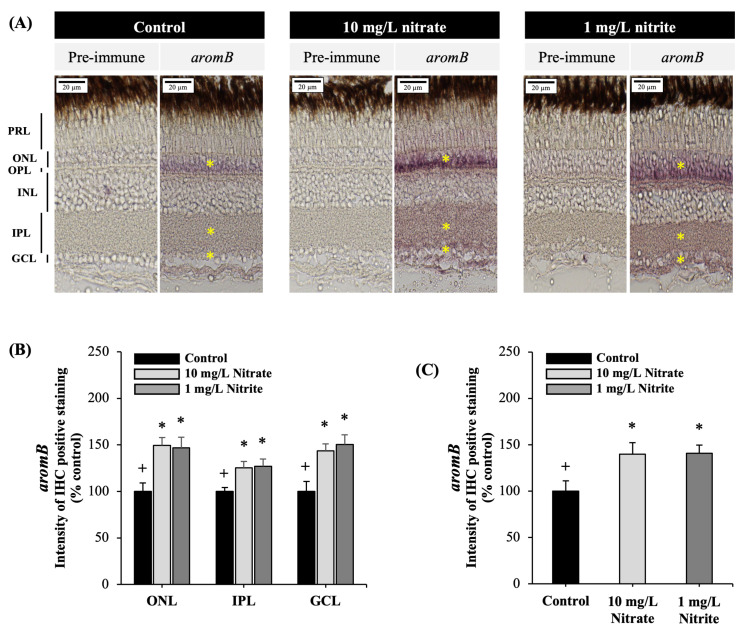
Brain aromatase (*aromB*) immunohistochemistry staining in adult zebrafish retina. Immunohistochemistry staining for *aromB* was performed on adult zebrafish retina exposed to control, 10 mg/L nitrate, and 1 mg/L nitrite. (**A**) Representative image of zebrafish retina with *aromB* positive staining exposed to control, 10 mg/L nitrate, and 1 mg/L nitrite. The yellow asterisk indicates the positive area of *aromB* protein in the retina layer. (**B**) The intensity of IHC positive staining in the outer nuclear layer (ONL), inner plexiform layer (IPL), and ganglion cell layer (GCL). (**C**) The overall intensity of IHC positive staining across all retina layers (ONL, IPL, and GCL) following exposure to nitrate and nitrite. Each value is expressed as the mean ± SD (*n* = 5). Different symbols (+, *) in each graph indicate significant differences (*p* < 0.05). Similar experiments were replicated three times.

In the adult zebrafish retina, immunolabeling with an antibody targeting *aromB* demonstrated significant presence of positive areas in the ONL and IPL, with reactivity also detected in the GCL (Figure 5A). The results of the study indicated that exposure to nitrate and nitrite significantly increased *aromB* protein expression intensity in the ONL, IPL, and GCL (Figure 5B), as well as overall retina layers (Figure 5C).

## 4. Discussion

We have demonstrated that exposure to nitrate and nitrite, at levels close to the safety limits recommended for drinking water by the US EPA and WHO (10 mg/L NO_3_-N and 1 mg/L NO_2_-N) [6], has negative effects on the visual system of adult zebrafish. The ecological and physiological effects of nitrate and nitrite pollution are highlighted by the fact that these concentrations are indicative of levels that are significant to the environment [8]. Surface water has been shown to contain nitrate levels ranging from 1 to 30 mg/L NO_3_-N [30]. In certain instances, such as in ponds and streams, nitrate levels might exceed 100 mg/L NO_3_-N [31]. Similar to our finding, the nitrate and nitrite concentration used here decreased tyrosine hydroxylase expression and motor behavior in zebrafish [16]. Previous research on zebrafish has demonstrated that elevated levels of nitrate or nitrite, ranging from 100 to 300 mg/L, are associated with developmental abnormalities, growth suppression, and high mortality [13], as well as alterations in brain metabolome and behavior [17]. These findings emphasize the complex and potentially harmful effects of nitrate and nitrite pollution on aquatic organisms, even at levels considered within regulatory safety limits [6].

The OMR test has been crucial in identifying visual impairments caused by genetic mutations and toxin exposures in adult zebrafish [32,33]. OMR tests have also assessed environmental contaminants’ effects on vision. Exposure to heavy metals, pesticides, and other toxic compounds impairs zebrafish OMR, demonstrating their adverse effects on the visual system [34]. Our study demonstrated that exposure to nitrate and nitrite resulted in reduced swimming behavior in response to the positive directions of the OMR stimulus. Similarly, previous research reported that zebrafish exhibited reduced swimming behavior in response to OMR stimuli after exposure to H_2_O_2_ [27]. These findings indicate that exposure to nitrate and nitrite may contribute to abnormal visual behavior, suggesting a potential association between such exposure and visual impairments. Previous studies conducted by Pelkowski et al. (2011) [35] and Richendrfer and Créton (2013) [36] found that zebrafish exhibited avoidance behavior in response to visual stimuli. Additionally, fish have been shown to swim away from a computer-generated bouncing ball, further indicating their tendency to display avoidance behavior [37]. Our results showed that exposure to the animated stimulus caused significant movement away from the moving grating in the control group, whereas the exposed group showed no significant change. These findings suggest that defects in OMR and avoidance behavior following exposure to nitrate and nitrite indicate major visual impairment. The exposed zebrafish were unable to properly align with moving visual stimuli or execute avoidance behaviors, indicating that nitrate and nitrite may disrupt the normal functioning of the visual system.

The eye, much like other organs, maintains a high degree of structural conservation between zebrafish and mammals [38]. The adult zebrafish retina exhibits distinct layers as visualized through H&E staining. These layers include the PRL and ONL, which contains photoreceptor cell bodies that synapse in the OPL alongside bipolar and horizontal cells. The INL contains horizontal, bipolar, and amacrine cell bodies, while the GCL contains the cell bodies of retinal ganglion cells (RGC) [39]. Moreover, INL comprises the nuclei of bipolar, horizontal, amacrine cells, and Müller glial cells, playing a crucial role in transmitting information from photoreceptors to ganglion cells [40]. Müller glial cells within the INL contribute to maintaining retinal homeostasis. Additionally, the IPL facilitates synaptic connections between bipolar and amacrine interneurons’ axons and ganglion cells’ dendrites [39]. Reducing the thickness of the zebrafish retina can lead to significant visual impairment and dysfunction. Research indicates that reduced thickness in layers such as the PRL, ONL, INL and IPL is linked to impaired ocular development and visual function [41]. Specifically, a thinner INL can disrupt the transmission of visual information from photoreceptors to ganglion cells [40], while a thinner IPL can hinder the synaptic connections necessary for processing visual signals [39]. Genetic mutations affecting retina layer formation and maintenance in zebrafish have been linked to significant vision deficits [42]. Our study demonstrated that exposure to nitrate and nitrite significantly reduced retinal layer thickness. These structural changes can lead to compromised visual behaviors, emphasizing the critical role each retinal layer plays in maintaining overall visual health and functionality.

Exposure significantly reduced most of the retina layers accompanied with decreased expression of genes related to eye development. In vertebrates, *pax6* genes are critical transcription factors for lens, retina, and cornea development. *Pax6* is still highly expressed in the cornea, iris, and lens in adults, highlighting its importance in eye structure and function [43]. Mutations in the *pax6* gene can cause aniridia, a disorder characterized by absent or underdeveloped iris, nystagmus, foveal hypoplasia, and related complications such as cataracts, glaucoma, and corneal keratopathy. These conditions highlight the profound impact of *pax6* gene defects on visual health [44]. This study provides evidence that lowering the expression levels of *pax6a* and *pax6b* genes may result in a noticeable reduction in retinal thickness among adult zebrafish. This reduction in retinal thickness is linked to alterations in eye function, suggesting that *pax6* genes play a crucial role in maintaining the structural integrity and functionality of the zebrafish eye [45]. Decreased expression of *pax6* has been linked to impaired eye function [46], demonstrating the importance of *pax6* genes in regulating retinal development and their impact on eye health and visual abilities in adult zebrafish.

Increased levels of reactive oxygen species (ROS) may indicate an impaired antioxidant defense system, as seen through reduced activities of essential antioxidant enzymes like superoxide dismutase (SOD) and glutathione peroxidase (GPX), along with lower levels of the non-enzymatic scavenger glutathione (GSH) [47]. SOD is critical enzymes involved in ROS removal [48], while GSH is an essential intracellular antioxidant, especially in alveolar epithelial cells. Glutathione peroxidases, particularly GPx1, have been linked to the development and prevention of a variety of common and complex disorders [49]. The current study demonstrated that exposure to nitrate and nitrite led to a decrease in the expression of *sod1* and *gpx1a*, potentially leading to oxidative stress. It has been reported that oxidative stress caused visual defects in zebrafish by decreased the antioxidant gene expression [27]. These findings highlight the crucial role of strong antioxidant defenses in biological systems exposed to environmental stressors like nitrate and nitrite. These defenses protect cells, including those in the visual system, from oxidative damage, ensuring overall cellular health and function.

Exposure increased the expression of *nos2a* in the adult zebrafish eye, indicating elevated nitric oxide (NO) production. The harmful impacts of nitrate, influenced by NO, are increasingly acknowledged across diverse biological settings [18]. Many mechanisms, including ascorbic acid, xanthine oxidoreductase, deoxygenated hemoglobin, mitochondrial enzymes, and other pathways, reduce nitrate–nitrite to NO. Apart from the existence of bacterial reductase in animals [50], it has been recognized that xanthine oxidoreductase sequentially reduces NO_3_^−^ to NO_2_^−^ to NO in mammalian tissues [51]. Studies have demonstrated that nitrate can be converted to nitrite and subsequently to NO in the body, particularly under conditions of inflammation or stress [19]. Elevated levels of NO have been associated with pathological conditions such as cardiovascular diseases, neurodegenerative disorders, and inflammatory diseases [21,52]. Excessive production of NO, particularly by *nos2a* (inducible NOS, iNOS), can contribute to oxidative stress and inflammation in the eye [53]. In diseases including glaucoma, diabetic retinopathy, and age-related macular degeneration (AMD), where oxidative stress and neuroinflammation play significant roles, iNOS expression contributes to neuronal damage and disease progression, leading to impaired visual function [54,55]. Thus, our study findings suggest that exposure to nitrate and nitrite increased *nos2a* expression, potentially leading to elevated NO production, which could contribute to visual impairment through oxidative stress damage.

Excessive oxidative stress can cause cell death through necrosis or apoptosis. Apoptosis is crucial for cell renewal, immunity, embryonic growth, metamorphosis, and stress response [56], and serves as a defense mechanism against cell damage from diseases or harmful agents [57]. The Bcl-2 protein, particularly *Bcl-2a*, is anti-apoptotic and helps regulate apoptosis by inhibiting it, promoting cell survival [58]. In zebrafish, *tp53* is a key regulator of apoptosis, activating the apoptotic pathway in response to stress, damage, or genetic abnormalities [59]. Furthermore, *tp53* also interacts with other proteins, like Bcl-2 family members, to balance pro-apoptotic and anti-apoptotic signals, determining cell fate [60]. In our study, exposure to nitrate and nitrite increased the expression of *tp53* and *casp3*, while reducing the expression of the anti-apoptotic gene *bcl2a*. Research has shown that a reduction in the anti-apoptotic protein BCL-2 (encoded by the *bcl2a* gene) can contribute to the development or progression of breast cancer [61]. The research findings are consistent with previous studies that demonstrated how overexpression of zebrafish *tp53* or *casp3* can induce apoptosis in various cellular systems, including cultured fish cells and embryos [56], Rat1 [62], Xenopus XLT-15-11 [63], and HECK-293T cells [64]. These findings suggest that apoptosis was triggered, leading to impaired visual function in zebrafish. The activation of apoptosis pathways and subsequent impairment of visual function highlight the sensitivity of the zebrafish visual system to environmental stressors.

Moreover, estrogen (E2) plays a significant role in the visual system, with aromatase expressed in the retina and estrogen receptors (ERs) present in all retinal layers across various vertebrate species [65,66,67]. Estrogen is synthesized directly from testosterone by the enzyme aromatase, also known as estrogen synthase [68]. Zebrafish have two distinct aromatase-expressing genes: cyp19a encodes aromatase A (*aromA*). These are found primarily in the gonads, while cyp19b encodes aromatase B (*aromB*), which is expressed in neural tissues such as the brain and retina [69]. E2 exerts its effects through two intracellular estrogen receptors (*esr1*, *esr2a*, and *esr2b*), which act as ligand-activated transcription factors to regulate estrogen target genes [70]. Our study revealed that exposure to nitrate and nitrite increased the expression of estrogen receptors (*esr1*, *esr2a*, and *esr2b*) and *cyp19a1b* as well as brain aromatase protein, confirmed by IHC. Interestingly, immunostaining demonstrated elevated levels of *aromB* protein in critical retinal layers such as ONL, IPL, and GCL of zebrafish eye. Similarly, immunolocalization of steroidogenic enzyme and estrogen receptor were distributes in the ONL, IPL, and GCL of goldfish retina [66], as well as in adult rat retina [67,71]. Given the importance of E2 in the visual system, Changes in E2 levels due to aging or hormone therapies are linked to neurodegenerative retinal diseases and visual complications, impacting eye structure and function [72]. Therefore, alterations in estrogen signaling following exposure to nitrate and nitrite may potentially disrupt visual function in adult zebrafish, highlighting the vulnerability of the visual system to environmental influences on hormonal pathways. Further research is necessary to elucidate the specific mechanisms through which nitrate and nitrite interact with estrogen signaling pathways.

## 5. Conclusions

In summary, our study strongly indicates that exposure to nitrate and nitrite adversely affects the vision of adult zebrafish. This exposure resulted in impaired visual functions and noticeable alterations in retinal layers. Furthermore, exposure to these chemicals disrupted oxidative stress responses, apoptosis pathways, and estrogen signaling. Collectively, our findings underscore the detrimental effects of nitrate and nitrite exposure on the visual abilities of aquatic organisms, emphasizing the urgent need to address environmental factors contributing to visual impairments in aquatic species.

## Figures and Tables

**Table 1:** Water quality parameter in the treatments tank.

Treatments	Temperature (°C)	Ammonia (mg/L)	Nitrate (mg/L)	Nitrite (mg/L)	pH
Morning (Pre-feeding)					
Control	27.0 ± 0.46	0.04 ± 0.01	0.05 ± 0.02	0.05 ± 0.02	7,3 ± 0.33
10 mg/L nitrate	27.0 ± 0.41	0.04 ± 0.01	10.2 ± 0.70	0.07 ± 0.02	7.3 ± 0.39
1 mg/L nitrite	27.3 ± 0.60	0.05 ± 0.01	0.05 ± 0.02	1.1 ± 0.12	7.1 ± 0.49
Afternoon (Post-feeding)					
Control	26.6 ± 0.39	0.05 ± 0.02	0.06 ± 0.02	0.05 ± 0.02	7.3 ± 0.40
10 mg/L nitrate	27.0 ± 0.63	0.06 ± 0.02	10.3 ± 0.34	0.07 ± 0.02	7.2 ± 0.49
1 mg/L nitrite	26.9 ± 0.56	0.06 ± 0.01	0.06 ± 0.02	1.19 ± 0.24	7.1 ± 0.42

Note: There was no effect of nitrite or nitrate on ammonia levels, temperature, and pH. Additionally, feeding did not alter the concentration of these water parameters. Data are expressed as mean ± SD.

**Table 2:** Primer sequences.

Gene Name	Primer Sequence (5′-3′)	Accession Number
*pax6a* (paired box 6a)	F: CTCAAACAGAAGAGCGAAATGGA R: GAAGCTGCTGCTGATGGGTAT	XM_009297889.3
*pax6b* (paired box 6b)	F: CCTCCAGTCACATTCCCATCA R: AGCATTGAGCCTGTCGTGAA	NM_131641.1
*sod1* (superoxide dismutase 1)	F: GTCGTCTGGCTTGTGGAGTG R: TGTCAGCGGGCTAGTGCTT	NM_131294.1
*gpx1a* (glutathione peroxidase 1a)	F: GGCACAACAGTCAGGGATTA R: CAGGACGGACGTATTTCAGA	NM_001007281.2
*nos2a* (nitric oxide synthase 2a)	F: GGAGATGCAAGGTCAGCTTC R: GGCAAAGCTCAGTGACTTCC	XM005165296
*casp3* (caspase 3)	F: CCGCTGCCCATCACTA R: ATCCTTTCACGACCATCT	NM_131877.3
*tp53* (tumor protein p53)	F: GGGCAATCAGCGAGCAAA R: ACTGACCTTCCTGAGTCTCCA	NM_131327.2
*bcl2a* (BCL2 apoptosis regulator a)	F: AGGAAAATGGAGGTTGGGATG R: TGTTAGGTATGAAAACGGGTGGA	NM_001030253.2
*esr1* (estrogen receptor 1)	F: CCGGCCCTACACAGAGATCA R: AGCCAAGAGCTCTCCAACAACT	NM_152959.1
*esr2a* (estrogen receptor 2a)	F: CTGTGCCGTCTGCAGTGATT R: CGGCGGTTCTTGTCGATAGT	NM_180966.2
*esr2b* (estrogen receptor 2b)	F: TCCGACACCTCAGCAACAAA R: TTTCTGGGCTCTGTTGTCTGTCT	NM_174862.3
*cyp19a1a* (ovarian aromatase)	F: AGATGTCGAGTTAAAGATCC R: ACTCGTTGATAAAACTCTCC	NM_131154.3
*cyp12a1b* (brain aromatase)	F: GCAAATCGTACAGGAGATAC R: CGTCCAATGTTCAGGATTAG	NM_131642.2
*eef1a1* (elongation factor 1 alpha 1)	F: TGGTGGTGTCGGTGAGTTTG R: AAACGAGCCTGGCTGTAAGG	AY422992.1

## Data Availability

Data will be available from the corresponding author upon reasonable request.
